# Application of Network Pharmacology in the Treatment of Neurodegenerative Diseases with Traditional Chinese Medicine

**DOI:** 10.1055/a-2512-8928

**Published:** 2025-02-18

**Authors:** Qiang Chen, Guanghui Chen, Qianyan Wang

**Affiliations:** 1 Department of Pharmacy, Liyuan Hospital, Tongji Medical College, Huazhong University of Science and Technology, Wuhan, Hubei, China; 2 Department of Pharmacy, Renmin Hospital, Wuhan University, Wuhan, Hubei, China; 3 Liyuan Cardiovascular Center, Liyuan Hospital, Tongji Medical College, Huazhong University of Science and Technology, Wuhan, Hubei, China

**Keywords:** neurodegenerative diseases, traditional Chinese medicine, network pharmacology, therapeutic targets

## Abstract

In recent years, the incidence of neurodegenerative diseases, including Alzheimerʼs disease, Parkinsonʼs disease, Huntingtonʼs disease, and amyotrophic lateral sclerosis, has exhibited a steadily rising trend, which has posed a major challenge to the global public health. Traditional Chinese medicine, with its multicomponent and multitarget characteristics, offers a promising approach to treating neurodegenerative diseases. However, comprehensively elucidating the complex mechanisms underlying traditional Chinese medicine formulations remains challenging. As an emerging systems biology method, network pharmacology has provided a vital tool for revealing the multitarget mechanisms of traditional Chinese medicine through high-throughput technologies, molecular docking, and network analysis. This paper reviews the advancements in the application of network pharmacology in treating neurodegenerative diseases using traditional Chinese medicine, analyzes the current status of relevant databases and technological methods, discusses the limitations, and proposes future directions to promote the modernization of traditional Chinese medicine and the development of precision medicine.

## Introduction

Network pharmacology is an emerging discipline initially proposed by the British pharmacologist Andrew L. Hopkins in 2007 1 . This discipline integrates systems biology, network biology, computational simulation, and chemoinformatics to construct a “drug–component–target–disease” network model, aiming to reveal the multidimensional therapeutic mechanisms of complex diseases. Network pharmacology enhances the multitarget and multi-pathway analysis, which is particularly well suited for research on traditional Chinese medicine (TCM), aligning with the multicomponent and multi-pathway therapeutic characteristics of TCM, and compensating for the limitations of traditional single-target pharmacological research 2 , 3 .

**Fig. 1:**
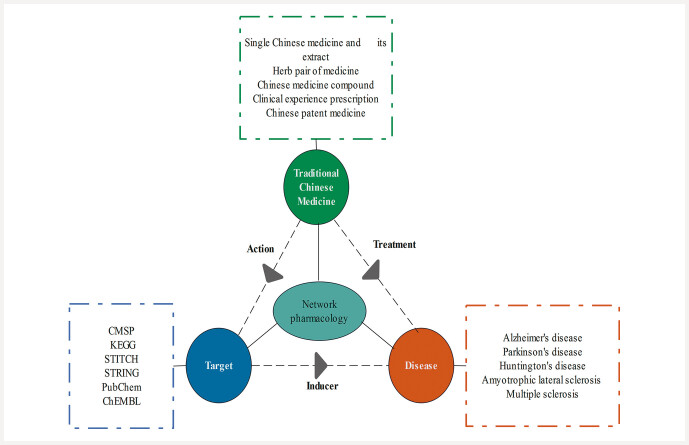
Relationship between digestive diseases and network pharmacology.

Neurodegenerative diseases include Alzheimerʼs disease (AD), Parkinsonʼs disease (PD), Huntingtonʼs disease (HD), and amyotrophic lateral sclerosis (ALS) 4 . These diseases can gradually result in the degeneration of central nervous system functions, which is manifested in a decline in memory, motor control, and cognitive ability 5 , 6 . For example, AD is identified as the leading cause of dementia in the elderly, while PD is primarily characterized by motor dysfunction 7 . Current treatments mainly focus on slowing down disease progression with medication, while their efficacy is limited and cannot cure any of these diseases 8 . For instance, AD treatments mainly focus on slowing down cognitive decline, but their long-term effects remain uncertain, and side effects are common 9 . Emerging therapies such as stem cell therapy and gene therapy have aroused wide attention; meanwhile, lifestyle factors including diet, exercise, and sleep are increasingly recognized for their roles in preventing and managing these diseases 10 , 11 . With the global population aging, the incidence of neurodegenerative diseases is rising significantly, especially in developed countries 12 , 13 . Although the current treatments can delay disease progression to some extent, the complexity of these diseases presents major clinical challenges. Therefore, the search for more effective treatments has become an urgent priority. The multitarget and multi-pathway analysis methods of network pharmacology provide novel possibilities for studying neurodegenerative diseases. Numerous effective components and formulations in TCM have already been illustrated through network pharmacology to show their mechanisms of action in treating these diseases 14 . The relationship between digestive diseases and network pharmacology is shown in Fig. 1 .

## Commonly Used Network Pharmacology Databases

### Traditional Chinese medicine systems pharmacology database and analysis platform

Traditional Chinese medicine systems pharmacology (TCMSP) is a systems pharmacology database developed for TCM research, integrating chemical information, biological activities, targets, and pharmacokinetic parameters of TCM components 16 . Covering 499 herbal medicines, 29 384 compounds, and 3311 targets, it is widely used for screening multicomponent and multitarget drugs and exploring mechanisms of action 17 , 18 . TCMSP offers tools for identifying potential effective components and predicting their absorption, distribution, metabolism, and excretion (ADME) properties 19 , 20 . For example, active components in Scrophularia ningpoensis were found to modulate 40 targets related to *β* -amyloid and tau proteins in AD 21 . Effective components in Suanzaoren decoction were identified as modulating pathways associated with insulin resistance and inflammation, improving AD-related diabetes symptoms 22 . Data from TCMSP also revealed that BM25 (Ershiwuwei Shancorawan) exhibited neuroprotection and anti-inflammatory activities by regulating multiple targets, reducing *β* -amyloid and tau protein accumulation, and delaying cognitive decline in AD 23 . In addition, TCMSP was used for analyzing the mechanisms of Jianpi Huatan Quyu decoction in treating chronic heart failure, exhibiting its cardiovascular protection and anti-inflammatory effects by regulating several pathways 24 ( Table 1 ).

**Table:** **Table 1** Public databases related to TCM network pharmacology.

Type	Name	Description	Website of database or tool
TCM-related database	TCMSP	A database that can retrieve and screen for the key TCM compounds and target information.	http://tcmspw.com/tcmsp.php
Drug-related database	KEGG	A database for understanding the high-level functions and utilities of biological systems, such as cells and organisms.	https://www.genome.jp/kegg/
Drug-related database	STITCH	A database containing the known and predicted chemical compounds and protein interactions.	http://stitch.embl.de/
Target-related database	STRING	A database of known or predicted interactions between proteins.	https://string-db.org/
Drug-related database	PubChem	A free chemistry database maintained by the National Center for Biotechnology Information (NCBI), which provides information on chemical molecules and their activities in biological assays.	https://pubchem.ncbi.nlm.nih.gov/
Drug-related database	ChEMBL	A database integrating bioactive substances that possess drug-like characteristics.	https://www.ebi.ac.uk/chembl

### The Kyoto encyclopedia of genes and genomes database

Kyoto encyclopedia of genes and genomes (KEGG) is an integrative bioinformatics database encompassing gene, protein, disease, and metabolism data. Through investigating signal transduction and metabolic pathways, KEGG helps researchers reveal drug mechanisms in complex diseases. In addition, it is widely used in network pharmacology to investigate how TCM components regulate sophisticated pathways. KEGG analysis suggested that active components of Asparagus officinalis mitigated fluorosis-induced oxidative stress and inflammation, protecting brain cells through antioxidative and anti-inflammatory mechanisms 25 . Mahboob et al. employed KEGG to identify AD-related genes and pathways, highlighting their roles in apoptosis, neuroinflammation, and oxidative damage 10 . These findings demonstrate the critical role of KEGG in clarifying mechanisms underlying neurodegenerative diseases.

### The search tool for interactions of chemicals database

Search tool for interactions of chemicals (STITCH) integrates interaction data between genes, proteins, and chemicals, including over 2.6 million proteins and 30 000 small molecules from 1133 species 26 , 27 . Through the combination of computational predictions, experimental validation, and literature-based associations, STITCH provides compound-target interaction networks, helping researchers explore multitarget mechanisms of chemicals. This database is widely used in drug discovery and target screening, allowing researchers to predict compound effects in multitarget diseases by examining interactions with multiple protein targets. STITCH identified AD-related differentially expressed genes (DEGs) and several key pathways through enrichment analysis 28 . Olatunde et al. employed STITCH to construct protein-compound networks, showing multitarget, multicomponent mechanisms of TCM in treating complex diseases including diabetes and cancer 29 . STITCH offers Application Programming Interfaces (API) and interactive network views for investigating protein-chemical interactions in biological processes 30 . These studies emphasize the value of STITCH in illuminating the multitarget mechanisms of TCM.

### The search tool for the retrieval of interacting genes/proteins database

Search tool for the retrieval of interacting genes/proteins (STRING) is a database concentrates on protein-protein interactions, integrating experimental verification and predictive data to provide insights into functional and physical relationships between proteins. It has been widely applied to explore drug targets and construct protein regulatory networks. Using STRING, researchers can identify direct and indirect protein interactions and examine their potential impacts on disease development, making it especially significant for research on multitarget disease mechanisms. In network pharmacology research, STRING is usually used for analyzing interaction networks between effective TCM components and disease-related proteins. By constructing multitarget protein interaction networks, STRING contributes to uncovering the multidimensional regulatory mechanisms of TCM in complex disorders, further elucidating the associated TCM mechanisms and potential therapeutic targets. For example, in a study on Andrographis paniculata for the treatment of AD, the authors utilized both STITCH and STRING databases to construct protein-compound interaction networks, identifying key AD-related pathways, including NF- *κ* B and PI3K-Akt. Experimental verification showed that effective compounds from A. paniculata significantly lowered inflammatory proteins, including PTGS2 (COX2) and BACE1, consequently suppressing the progression of AD 31 . This study underscores the importance of STRING in unveiling the multitarget mechanisms of TCM.

### PubChem

PubChem is a public chemical information database established by the U. S. National Institutes of Health. It integrates chemical structures, biological functions, properties, and pharmacological effects, covering millions of compounds and experimental bioactivity data. As one of the largest open-access chemical database **s** , PubChem exerts an essential role in drug discovery, compound screening, and bioactivity analysis, especially for drug development and repositioning. In network pharmacology, PubChem is widely used to obtain detailed chemical information and analyze interactions with disease-related genes or proteins in combination with other databases. In addition, it enables large-scale compound screening and evaluation of their therapeutic potential. For example, in an AD study, PubChem was used to screen AD-related compounds, and network pharmacology revealed compound-target gene interactions, exhibiting their role in reducing neuroinflammation and mitigating neural injury 32 . Similarly, PubChem was used to identify ALS-related compounds and investigate their impacts on ALS pathology 33 . These studies underscore PubChemʼs importance in comprehending complex disease mechanisms.

### ChEMBL

ChEMBL, developed by the European Bioinformatics Institute, concentrates on bioactive compounds and offers extensive data on drugs and their targets 34 . With millions of bioactivity data entries for small molecules, it is widely used for drug discovery and target prediction, enabling researchers to explore drug-target interactions and screen potential drug candidates. In network pharmacology, ChEMBL provides bioactivity data for compounds and protein targets, supporting the analysis of interactions between small molecules or TCM components and disease-related genes. This helps uncover multitarget TCM mechanisms and advances network pharmacology-based drug development. ChEMBL also promotes research on neurodegenerative diseases by exploring multitarget compound bioactivity, particularly through datasets of animal disease models and phenotypic endpoints for translational medicine research 35 .

## Application of Network Pharmacology in Traditional Chinese Medicine Research

### Network pharmacology research techniques

Network pharmacology combines the updated approaches like high-throughput technology, molecular docking, and systems biology to assist researchers in analyzing the multitarget, multi-pathway TCM mechanisms. Among them, high-throughput technology can process a variety of samples concurrently, rapidly producing massive biological data, like protein interactions, gene expression profiles, or metabolite changes. It has been frequently adopted for screening effective TCM components and the possible associations with disease targets. For instance, in a study concerning AD, high-throughput technology was used to analyze different TCM compounds, and finally, multiple effective components with therapeutic effects were identified 36 . Molecular docking can simulate the drug molecule-target protein binding patterns and predict the corresponding interactions. It contributes to validating binding sites and action mechanisms of TCM components with corresponding targets. For example, in a study regarding PD, molecular docking was used to discover the effective binding of ligustrazine to proteins associated with the PI3K-Akt pathway, demonstrating the neuroprotection 37 . Systems biology integrates multilevel biological data (including proteins, genes, and metabolites) to illustrate interactions inside the complicated biological networks. In network pharmacology, it is applied to analyze the multidimensional mechanisms of TCM formulations. The combined application of these techniques can help researchers uncover the multitarget mechanisms of TCM formulations in treating complex diseases, transforming complex network interactions into visualized networks, and facilitating the modernization of TCM and the development of precision medicine.

### Steps in network pharmacology research and construction of biological networks

**Fig. 2:**
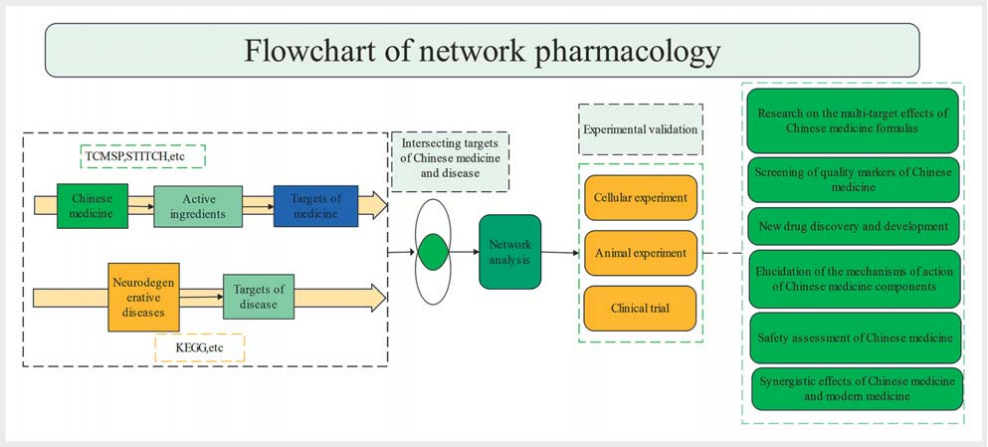
Flowchart of network pharmacology analysis.

The typical steps in network pharmacology research are as follows: at first, bioactive components are screened from TCM. Second, the potential targets of these components are identified using relevant databases. Third, a “drug–component–target–disease” network model is constructed based on these targets and components, and network analysis is performed to identify key regulatory nodes 38 . Finally, these results should be validated by conducting biological experiments, aiming to ensure the reliability of the research. The construction and analysis of the network model are central to the research process, as they can reveal the complex mechanisms of TCM formulations and provide theoretical support for new drug development. Fig. 2 shows the flowchart of network pharmacology analysis.

### Applications of network pharmacology

Through systematic analysis using network pharmacology, researchers can predict new indications for TCM beyond its therapeutic effects. This process depends on the constructed biological networks. Relevant signaling pathways and targets can be identified. For instance, by examining the associations of TCM effective components with disease pathways, the possible therapeutic efficacy of TCM can be predicted under the novel pathology condition, which provides important support for new indication discovery 39 . Researchers can explore the multitarget effects of TCM formulations, screen for quality markers of TCM, further elucidate the mechanisms of TCM components, assess the safety of TCM, and explore the synergistic effects between TCM and modern drugs. These research directions can comprehensively enhance the scientific foundation and clinical application value of TCM, thereby promoting the modernization of TCM.

Finally, these results need to be validated through biological experiments to ensure the reliability of the study. Experimental validation is an indispensable part of network pharmacology research, as the results predicted by models are obtained solely through computational tools. Their accuracy and biological relevance need to be further confirmed. Common validation methods include cell experiments, animal studies, and molecular biology techniques. For example, in a study performed on the inhibitory potential of Lasunadya Ghrita (LG) extract against *β* -amyloid aggregation in AD, the anti-AD potential of the water extract of Lasunadya Ghrita (LGWE) was explored. However, an insufficient logical connection between network pharmacology and experimental validation in the antioxidant mechanism existed. Although the study emphasized that LGWEʼs complex components may regulate antioxidant pathways (such as Nrf2, HO-1, SOD1) through multiple targets, it did not systematically employ network pharmacology tools to predict specific target genes and pathway associations, causing a lack of solid theoretical foundation 40 . While the experiments demonstrated the phenomenon that LGWE inhibited *β* -amyloid-induced reactive oxygen species generation, deeper verification on predicted targets, such as Nrf2 or mitochondrial dysfunction, was not conducted, therefore leaving the mechanistic study at a superficial level. As a key tool for multitarget therapy, network pharmacology predictions require experimental validation to close the loop, otherwise, logical gaps may occur. The lack of network pharmacology integration and the limitations of experimental validation in this study weakened the comprehensive understanding of LGWEʼs antioxidant mechanisms. Moreover, research should integrate target prediction and validation tools, clarify the relationship between active components and antioxidant pathways, as well as supplement experiments related to antioxidant pathways to enhance the logical integrity and practical utility of the study.

## Application of Network Pharmacology in Neurodegenerative Diseases

### Application of traditional Chinese medicine in Alzheimerʼs disease

AD is a major neurodegenerative disorder affecting the elderly population globally, with the primary symptoms including memory loss, cognitive decline, and abnormal behavior. Approximately 50 million people globally suffer from dementia, and 60 – 70% of them develop AD. The number is expected to reach 100 million by 2050 41 . With the acceleration of global aging, the incidence of AD exhibits a significantly increasing trend, making it urgent to develop new treatment methods 36 . The pathological mechanisms of AD are complex and are suggested to involve *β* -amyloid aggregation, neuroinflammation, oxidative stress, and neuronal apoptosis 42 , 43 . Although acetylcholinesterase inhibitors and N-methyl-D-aspartate (NMDA) receptor antagonists are currently the main therapeutic agents for the treatment of AD, they only temporarily alleviate symptoms and are accompanied by side effects, without halting disease progression 44 . Therefore, the develop of multitarget treatments for AD is urgently needed. Recently, due to its multicomponent and multitarget characteristics, TCM has attracted widespread attention in AD treatment. Through network pharmacology analysis, researchers predicted the potential active compounds and targets in the Hengqing II formula for treating AD, exerting anti-inflammatory, antioxidant, and neuroprotective effects mainly by regulating signaling pathways such as PI3K-Akt, NF- *κ* B, and MAPK 45 . In another study, network pharmacology analysis was used to identify 30 candidate targets related to AD for the compound paeoniflorin, which were enriched into the oxidative stress and inflammation pathways. Moreover, the key targets identified included Nrf2 (encoded by NFE2L2) and TLR4, which were closely associated with oxidative stress and neuroinflammation. *In vivo* experiments also confirmed the neuroprotective effects of paeoniflorin on AD 46 . Network pharmacology analysis was conducted to identify 15 active compounds and 135 potential targets in the QZZD (Qin-Zhi-Zhu-Dan) formulation. *In vitro* experiments demonstrated the impact of this formulation on alleviating neuroinflammation and slowing down neuronal death, exerting significant neuroprotective effects against AD 47 . Qiu et al. used network pharmacology and molecular docking techniques to explore the potential mechanisms of *Acorus calamus* in AD treatment, identifying four active components associated with multiple AD-related targets (e.g., AKT1 and MAPK14) that regulated the oxidative stress and anti-inflammatory pathways. Molecular docking further validated the binding abilities of these components to their targets, indicating the neuroprotective potential of *A. calamus* in AD 48 . Soleimani Zakeri et al. constructed protein-protein interaction networks to explore the gene complexes in AD and proposed drug repositioning candidates. Through bioinformatics analysis, they identified several key AD-related gene pathways and recommended two new potential treatments, namely, raloxifene and gentian violet 49 . These studies demonstrate the scientific significance of TCM in AD treatment, and the application of network pharmacology combined with *in vitro* experimental validation provides a better understanding of the multicomponent, multitarget, and multi-pathway therapeutic properties of TCM. In addition, this offers a theoretical basis and support for future drug research and development for AD.

### Application of traditional Chinese medicine in Parkinsonʼs disease

PD is a common neurodegenerative disease that primarily affects the elderly. Globally, the incidence of PD significantly increases with age, and it affects approximately 1% of people aged over 60 years and up to 3% of people over 80 years old 50 . With the global population aging, the incidence of PD is expected to rise further in the future 51 . The pathological mechanisms of PD are the progressive loss of dopaminergic neurons in the substantia nigra and the formation of Lewy bodies, which may be caused by abnormal aggregation of *α* -synuclein 52 . Mitochondrial dysfunction, oxidative stress, neuroinflammation, and immune responses are key factors for the pathogenesis of PD 53 . Existing treatments, including dopamine replacement therapy (like levodopa), dopamine receptor agonists, and MAO-B inhibitors, can temporarily relieve motor symptoms. However, as the disease progresses, the drug efficacy gradually decreases, and long-term use of these drugs may lead to side effects, including motor complications 54 . Against this backdrop, developing the multitarget treatments that can delay neurodegeneration and protect dopaminergic neurons has become a research focus. Network pharmacology can provide vital support for multitarget, multi-pathway TCM treatment for PD. For example, Liu et al. used network pharmacology to identify effective components and PD-related targets in Tianqi Pingchan granules (TPG), discovering that these targets were mainly related to inflammatory responses through Gene Ontology (GO).

and KEGG pathway analyses. *In vivo* experiments showed that TPG could regulate gut microbiota, reduce peripheral inflammatory factors, inhibit microglial activation, alleviate neuroinflammation, and delay PD progression 55 . Wu et al. utilized transcriptome sequencing to identify DEGs between PD patients and healthy individuals. Combined with network pharmacology analysis, the authors determined the relationship between active compounds in *Cordyceps sinensis* capsules and PD targets and constructed a “compound–disease–target” network. Quercetin and kaempferol, identified as the major anti-PD effective components, were found to exert anti-neuroinflammatory and neuroprotective effects by binding to key targets, including matrix metalloproteinase-9 (MMP9) 56 . Another study employed network pharmacology combined with molecular docking analysis to analyze 97 effective components of ginseng and 168 potential PD-related targets. Based on their experimental results, these targets were involved in regulating biological processes such as cellular metabolism and apoptosis, while further analysis indicated that the MAPK signaling pathway and AGE-RAGE signaling pathway might be associated with the treatment mechanisms for PD 57 . Wu et al., through network pharmacology, identified that the IL-17 signaling pathway and neuroactive ligand-receptor interaction were the key targets. *In vivo* experiments showed that DiHuangYin (DHY) reduced peripheral inflammation and central nervous system inflammation, thus protecting neurons 58 . Lin et al., by using network pharmacology and molecular docking techniques, revealed the main effective components of Liuwei Dihuang Wan, including quercetin, *β* -sitosterol, and kaempferol, which were closely related to multiple targets (such as AKT1, VEGFA, and IL6). Experimental studies indicated that Liuwei Dihuang Wan had potential neuroprotective effects against PD via the multitarget, multi-pathway mechanisms, particularly in regulating neuronal death, oxidative stress, and inflammatory responses 59 . The above results scientifically confirm the multitarget, multi-pathway mechanisms of TCM in PD treatment. Integrating network pharmacology and *in vitro* experiments can shed novel light on the complicated mechanisms and establish a solid basis for developing and applying PD-related drugs.

### Application of traditional Chinese medicine in Huntingtonʼs disease

HD refers to an uncommon inherited neurodegenerative disease. Its global incidence is approximately 5 – 10/100 000 individuals, and it usually occurs in middle age 60 . HD shows the representative characteristics of involuntary movements, psychiatric symptoms, and cognitive decline. The pathology includes mutant huntingtin protein aggregation, resulting in excessive neuronal apoptosis and serious central nervous system injury 61 . There are few treatments for HD, which mainly focus on managing its symptoms, including applying antipsychotics in controlling psychiatric and motor symptoms. Nevertheless, these treatments cannot prevent disease progression and may lead to significant adverse reactions 62 . Therefore, developing treatments that can delay and reverse HD pathology is becoming the research focus. Network pharmacology offers a novel research direction to explore whether TCM can be applied to treat HD. Using the multitarget, multi-pathway methods can uncover the effects of TCM components on regulating inflammation and neuroprotection. The HDNetDB database combines HD-related gene expression profiles and molecular interactions, contributing to exploring the complicated molecular mechanisms underlying HD and accelerating the systematic study of HD 63 . For example, network pharmacology studies have illustrated interactions among several TCM effective components and HD-related biological targets, especially those related to apoptosis and neuroprotection pathways like PI3K/Akt, NF- *κ* B, and MAPK. Molecular docking and Quantitative Structure-Activity Relationship (QSAR) models can be used to predict the possible effects of TCM components in treating HD, aiming to provide a theoretical foundation for the multitarget application in the clinic 64 . Likewise, according to network pharmacology research, natural compounds can regulate critical targets of HD, particularly via the gene-protein interaction networks, which can investigate NF- *κ* B and PI3K/Akt pathways within neuroinflammation. The above compounds are verified with neuroprotection through molecular dynamics simulations and *in vitro* experimental verification 65 . For instance, network pharmacology research suggests interactions of several TCM effective components with HD-related biological targets, especially by establishing the drug–target–disease networks, contributing to exploring the efficacy of TCM chemical components in treating diseases by modulating several pathways, including MAPK and PI3K/Akt 66 . Therefore, integrating network pharmacology with *in vitro* experimental verification can validate the multicomponent, multitarget TCM mechanisms in treating HD. The method provides a novel direction for analyzing the effect of TCM in treating HD and lays a solid foundation for drug development and clinical application 67 .

### Application of traditional Chinese medicine in amyotrophic lateral sclerosis

ALS represents an uncommon neurodegenerative disorder, whose incidence is around 1.9/100 000 individuals annually in the world. Patients usually die in 2 – 5 years after being diagnosed 68 . It is pathologically characterized by progressive motor neuronal degeneration, which causes muscle weakness and atrophy, finally leading to respiratory failure-related death 69 . ALS has a complicated etiology that involves different pathological processes, like mitochondrial dysfunction, glutamate toxicity, neuroinflammation, and oxidative stress 70 . At present, although there are few treatments available for ALS, and just two drugs are approved by the FDA (riluzole and edaravone) for delaying its progression, they can achieve limited therapeutic effects and are incapable of reversing neuronal injury 71 . Therefore, it is vital to search for multitarget treatments. Network pharmacology provides novel avenues for the application of TCM in treating ALS. For example, Li et al. employed network pharmacology for analyzing the multitarget, multi-pathway mechanisms of Bushen Jianpi (BSJP) formulation in mitigating ALS symptoms like muscle atrophy, hypoxia-related conditions, and oxidative stress. As reported in a later randomized, double-blind, multicenter clinical study, BSJP significantly improved lung capacity and muscle strength among ALS cases, and delayed ALS progression, with no significant adverse reactions. This formulation may bring benefits by modulating the level of vitamin D3 in serum and recovering renal and hepatic functions 72 . Lin et al. analyzed the multitarget mechanisms of effective components in BSJP using network pharmacology analysis. Their findings suggest that effective components like kaempferol and quercetin were vital for neuroprotection by modulating mitochondrial activity, anti-inflammatory response, and oxidative stress. Clinical studies indicated the therapeutic effect of this formulation on mitigating ALS symptoms, especially for alleviating neuronal injury and delaying ALS progression 73 . Through molecular docking and network pharmacology analyses, the possible mechanisms by which nux vomica-treated ALS were explored. The main components were examined and shown to mitigate oxidative stress and neuroinflammation in ALS through pathways such as Ras, MAPK, and PI3K-Akt. Moreover, *in vitro* experiments demonstrated that nux vomica components showed great affinity to key targets, demonstrating that nux vomica displayed neuroprotection in ALS and might be developed as an anti-ALS treatment 74 . Using molecular docking and network pharmacology analyses, Li et al. 75 revealed that the major components of *Rehmannia glutinosa* , including glutathione and phytosterols, interacted with targets such as PTGS2, ESR1, and PPARG to regulate neuroprotection and oxidative stress pathways, especially via the estrogen receptor pathway. This reduced glutamate toxicity and neuroinflammation, suggesting the potential of *R. glutinosa* in the treatment of ALS 75 . Network pharmacology and molecular docking techniques were used to explore the therapeutic potential of the TCM “ *R. glutinosa* ” in ALS. This study revealed its possible therapeutic mechanisms by analyzing the interactions of the effective components of *R. glutinosa* with different ALS-related targets and signaling pathways 76 . Noor et al. demonstrated that constructing the multilevel networks between drug components, targets, and diseases using network pharmacology helped reveal the multitarget properties of herbs. This multitarget, multi-pathway feature was especially vital for the treatment of multifactorial diseases such as ALS. The study also highlighted the immense potential of TCM and Ayurvedic herbs in treating complex diseases, including ALS 77 . Another study introduced network pharmacology as a new “green” strategy, and demonstrated how predicting the behaviors of metabolites revealed the mechanisms of natural products as drug candidates. Through the combination of omics data, computational modeling, and chemical biology, this multidisciplinary approach contributed to elucidating the multitarget actions of drugs and guided drug discovery, especially in complex multicomponent natural medicines, revealing their potential in ALS treatment 78 . Collectively, these studies demonstrate the significant multitarget, multi-pathway effects of TCM in treating ALS. The application of network pharmacology combined with *in vitro* experiments can provide a scientific basis for fully uncovering the therapeutic mechanisms of TCM and open novel avenues for developing new drugs for ALS treatment.

### Application of traditional Chinese medicine in multiple sclerosis

Multiple sclerosis (MS) is a chronic central nervous system disease, which primarily affects young adults, especially women. Approximately 2.5 million people worldwide suffer from MS. The disease is characterized by demyelination within the central nervous system, causing various neurological dysfunctions, including motor impairment, sensory abnormalities, and cognitive decline 79 . The pathogenesis of MS is complex, involving immune system abnormalities, neuroinflammation, and axonal damage 80 . Existing treatments for MS mainly depend on immunomodulatory drugs such as interferon- *β* and corticosteroids. While these drugs can delay disease recurrence, they cannot stop its progression, and long-term use may cause severe side effects 81 . Therefore, developing multitarget therapeutic strategies, particularly those based on natural compounds, has been a research focus. Noor et al. constructed a drug–target–disease network, finding that the key effective components of Yishen Daluo decoction included quercetin and kaempferol. Protein-protein interaction network analysis revealed that IL-6 and AKT1 were the key targets. *In vitro* experiments suggested that Yishen Daluo decoction reduced lipopolysaccaride-induced inflammatory responses, revealing its potential therapeutic mechanisms in MS 82 . To sum up, integrating network pharmacology and *in vitro* experimental verification can efficiently discover TCM targets for MS treatment, and provide the targeted treatments. The integrated method illustrates the TCM mechanisms in MS treatment and establishes a solid foundation for individualized treatments and drug development. The network pharmacology of TCM in treating neurodegenerative diseases is presented in Table 2 .

**Table:** **Table 2** Network pharmacology of TCM in treating neurodegenerative diseases.

Reference	Disease	Formulas and herbs	Subjects	Main compounds	Main target	Main signaling pathway	Outcome
46	AD	Paeoniflorin	*In vivo* experiment	Paeoniflorin	Multiple targets	PI3K-AKT	Demonstrates neuroprotective effects
47	AD	Qin-Zhi-Zhu-Dan formula	Network pharmacology analysis	Multiple effective components	Multiple targets	PI3K-AKT	Enhances neuroprotective effect of the formulation
48	AD	Arisaema Rhizome	Molecular docking in network pharmacology	Active compounds of Arisaema Rhizome	Multiple targets	Multiple pathways	Reveals the potential therapeutic effects of Arisaema Rhizome
49	AD	Drug repurposing strategy	Protein-protein interaction networks	Multiple drugs	Multiple protein networks	Protein interaction pathways	Identifies the potential therapeutic drugs for AD
55	PD	Tianma Pingchan granules	Rat model	Multiple effective components	Neural junction-related pathways	PI3K-AKT	Alleviates symptoms related to PD
56	PD	Jichuan decoction	Transcriptome analysis	Multiple effective components	Key genes	Critical signaling pathways	Reduces inflammation and neurodegenerative factors
57	PD	Ginseng	Neuron cell model	Ginseng extracts	Multiple targets	PI3K-AKT	Inhibits apoptosis and protects neurons
58	PD	Dihuang decoction	Animal model	Multiple active ingredients	Neuroprotective targets	NF- *κ* B	Shows significant neuroprotective effects
59	PD	Liuwei Dihuang pills	Neuron cell model	Components of Liuwei Dihuang pills	Multiple targets	Inflammation pathway	Reduces inflammation and inhibits apoptosis
63	HD	TCM compound formula	Network pharmacology analysis	Multiple compounds	Multiple targets	Inflammation pathways	Reduces inflammation and improves symptoms
64	HD	Novel TCM formula	Molecular docking and *in vitro* experiments	Multiple compounds	Multiple targets	Inflammation and neuroprotection pathways	Demonstrates significant neuroprotective effects
65	HD	Natural compounds from *Dictyostelium discoideum*	Neuronal cell model	Multiple compounds	Multiple targets	Apoptosis and inflammation pathways	Demonstrates significant cell protective effects
66	HD	Active components of TCM	Network pharmacology analysis	Multiple TCM components	Multiple targets	Neuroprotection and apoptosis pathways	Shows strong neuroprotective effects
72	ALS	Bu-Shen-Jian-Pi decoction	Neuron and muscle cell models	Multiple effective components	Multiple targets	Oxidative stress and inflammation pathways	Relieves hypoxia and muscle atrophy, improves motor function
73	ALS	Bu-Shen-Jian-Pi decoction	ALS rat model	Multiple components	Multiple targets	Neuroinflammation and oxidative stress pathways	Delays disease progression and improves motor function
74	ALS	Strychnos nux-vomica	Neuron cell model	Extract of Strychnos nux-vomica	Multiple targets	Neuroinflammation pathways	Demonstrates significant neuroprotective effects
75	ALS	Prepared rehmannia	Cell model	Extracts of prepared rehmannia	Multiple targets	Inflammation and apoptosis pathways	Inhibits apoptosis and inflammation significantly
81	MS	Yishen Daluo decoction	Immune cell model and animal experiments	Multiple components	Multiple targets	PI3K-AKT	Reduces inflammation and protects neurons
82	MS	*Nigella sativa*	Neuroinflammation model	Extract of *Nigella sativa*	Multiple targets	Immune pathways	Demonstrates strong anti-inflammatory effects and reduces inflammatory cytokines

## Limitations and Improvement Directions of Network Pharmacology

Although network pharmacology has shown significant potential in elucidating the complex mechanisms of TCM in treating neurodegenerative diseases, its development still faces the challenges. Firstly, existing databases suffer from outdated information and limited coverage, making it difficult to comprehensively reflect the multidimensional characteristics of Chinese herbal medicine. Future research should concentrate on dynamically updating and expanding databases to incorporate omics data such as genomics, proteomics, and metabolomics, while also enhancing data standardization to guarantee the integrity and consistency of compound information.

Secondly, most network pharmacology models are static and fail to simulate the dynamic processes of drugs *in vivo* , particularly in multicomponent TCM formulas where time-dependent effects and synergistic actions have not been fully explored. The introduction of dynamic pharmacokinetic models, time-series analysis, and multiscale modeling will help to uncover the metabolic pathways and dynamic synergistic effects of TCM components *in vivo* , therefore providing more accurate theoretical support for multitarget therapies.

Moreover, the variability of TCM formulations, which results from differences in origin, processing methods, and storage conditions, can lead to discrepancies in model predictions. Standardized analysis using chemical fingerprinting and metabolomics techniques, integrating variability data, and establishing dynamic adjustment mechanisms in models can effectively improve the reliability and applicability of predictions.

Insufficient experimental validation is another limiting factor. The lack of experimental support for computational predictions may restrict their clinical translation value. Combining efficient experimental systems such as organoid models, microfluidic chips, as well as cell experiments and animal models, can provide multilayered validation of network pharmacology predictions, providing strong support for practical applications.

Finally, personalized medicine is a vital direction for future development. Current models have not adequately considered the impact of individual genetic differences on responses to TCM treatments. Future research should integrate genomics and epigenetics to build personalized models, enabling the development of precise treatment plans that enhance efficacy and minimize adverse reactions. By integrating multi-omics data, dynamic modeling, and efficient experimental validation, network pharmacology is poised to further advance the modernization of TCM and precision medicine, thus offering innovative solutions for neurodegenerative diseases.

## Contributorsʼ Statement

Data collection: Q. Chen, G. H. Chen; Design of the study: Q. Chen, Q. Y. Wang; Statistical analysis: Q. Chen; Analysis and interpretation of the data: Q. Chen, G. H. Chen, Q. Y. Wang; Drafting the manuscript: Q. Chen, G. H. Chen; Critical revision of the manuscript: Q. Y. Wang.
